# Vitamin D supplementation in people with IBS has no effect on symptom severity and quality of life: results of a randomised controlled trial

**DOI:** 10.1007/s00394-021-02633-w

**Published:** 2021-07-30

**Authors:** Claire E. Williams, Elizabeth A. Williams, Bernard M. Corfe

**Affiliations:** 1 Molecular Gastroenterology Research Group, Department of Oncology & Metabolism, The Medical School, The University of Sheffield, Beech Hill Road, Sheffield, S10 2RX UK; 2 Department of Oncology and Metabolism, The Medical School, Healthy Lifespan Institute, The University of Sheffield, Beech Hill Road, Sheffield, S10 2RX UK; 3 Human Nutrition Research Centre, Faculty of Medical Sciences, Population Health Sciences Institute, Newcastle University, Newcastle, NE2 4HH UK

**Keywords:** Irritable bowel syndrome, Vitamin D, Vitamin D deficiency, Symptom management, Quality of life

## Abstract

**Purpose:**

Several small trials suggest a benefit of vitamin D supplementation in irritable bowel syndrome (IBS). The generalisability of these reports is limited by their design and scale. This study aimed to assess whether vitamin D supplementation improved IBS symptoms in a UK community setting.

**Methods:**

This was a randomised, double-blind, placebo-controlled study. Participants were recruited from the community in winter months between December 2017 and March 2019. 135 participants received either vitamin D (3,000 IU *p.d*.) or placebo for 12 weeks. The primary outcome measure was change in IBS symptom severity; secondary outcomes included change in IBS-related quality of life.

**Results:**

The participants were analysed on an intent-to-treat basis. 60% of participants were vitamin D deficient or insufficient at baseline. Although vitamin D levels increased in the intervention arm relative to placebo (45.1 ± 32.88 nmol/L *vs* 3.1 ± 26.15 nmol/L; *p* < 0.001). There was no difference in the change of IBS symptom severity between the active and placebo trial arms (− 62.5 ± 91.57 *vs* – 75.2 ± 84.35, *p* = 0.426) over time. Similarly there was no difference between trial arms in τhe change in quality of life (− 7.7 ± 25.36 *vs* – 11.31 ± 25.02, *p* = 0.427).

**Conclusions:**

There is no case for advocating use of vitamin D in the management of IBS symptoms. The prevalence of vitamin D insufficiency suggests routine screening and supplementation should be implemented in this population for general health reasons.

This trial was retrospectively registered with ISRCTN (ISRCTN13277340) on 24th April 2018 after recruiting had been initiated.

**Supplementary Information:**

The online version contains supplementary material available at 10.1007/s00394-021-02633-w.

## Introduction

Irritable Bowel Syndrome (IBS) is a highly prevalent functional bowel disorder, with estimates of numbers affected in westernised populations ranging widely, but often in the region of 10–15%[1], although this estimate has been revised to under 5% [2] with the introduction of revised ROME IV criteria for assessment [3]. It is characterised by chronically relapsing perturbed bowel habit, associated pain and sensitivity, and dissatisfaction with bowel movements[4]. Symptoms may be severe and significantly impact both social function and work, with predicted cost to the NHS in excess of £11 M *p.a*[5] and estimates of direct healthcare costs from £45-200 M in the UK [6], indirect costs are likely to be higher when the impacts of the condition on work are considered [*ibid*.]. The aetiology of IBS is not well-understood: infection, stress, dietary factors, impaired gut-brain signalling are all implicated, but none conclusively[7]. As a result, treatment is limited to symptom management. Pharmaceutical approaches include anti-spasmodic and anti-depressive drugs. Whole dietary approaches to symptom management include low-FODMAP diets and other exclusion-led approaches[8]. There is also interest in supplementation strategies, including probiotics, prebiotics[9] and recently glutamine supplementation[10]. What is unequivocal is that in all trials and approaches there is a heterogeneity of response (Williams & Corfe; manuscript in preparation); for patients, trial and error lead to restrictive behaviours in a form of personalised dietary management[11] although nutritional intake seems generally adequate[12]. The nature and impact of symptoms, coupled to lack of a clear treatment path, have associated impacts on mental health and well-being[13].

Vitamin D is a prohormone produced by epidermal photoconversion of 7-hydroxycholesterol to vitamin D_3_, followed by sequential hepatic, then renal, dihydroxylation to yield 25(OH) vitamin D then 1,25(OH) vitamin D[14]. The monohydroxylated form has a longer half-life and is usually used as a status marker. Low sunlight exposure through latitude, reduced mobility, or for cultural reasons is a risk factor for low vitamin D status[15]. Vitamin D is also obtained through diet and through supplementation. Low vitamin D status is a risk factor for poor bone health, with guidance on intake informed by reduced risk of fracture[16]. Nonetheless vitamin D is also implicated in non-skeletal pathologies[17]. From a gastroenterological perspective, the vitamin D receptor is strongly expressed in the colon[18]. Low vitamin D is a potential risk factor for colorectal carcinogenesis[19] and inflammatory bowel disease[20]. However, causal relationships between observed low vitamin D status in inflammatory conditions may be confounded by potential sequestration of the vitamin driven by inflammatory pathways[21].

Exploration of links between vitamin D status and IBS has arisen due to links between vitamin D and other colorectal pathobiologies. An untargeted analysis of mRNA from patients with IBS compared with controls suggested altered expression of serotonin update and metabolism pathways[22]. The same study showed reduced levels of TPH1 expression in IBS associated with vitamin D status, and went on to show with in vitro models that vitamin D treatment restored expression of EphA3 and CYP24A1 (vitamin D 24-hydroxylase) [22]. A case study[23] systematically collated patient reports of self-administration and suggested a potential benefit of vitamin D supplementation. Our review of vitamin D trials in management of IBS symptoms[24] noted that studies consistently reported prevalent vitamin D deficiency in participants with IBS, although there is inconsistency as to whether this is greater than in the general population (*ibid*.). Five RCTs have tested the effect of vitamin D in the management of IBS symptoms[25–29], with all reporting significant positive outcomes. However, four of these trials used bolus dosing (50,000 IU), one [27] (and potentially two—the dosing regime is ambiguous in [26]) with an effective dose above safe upper limit. Two trials used 6-week interventions[26, 27], which can obscure effect size relative to placebo in IBS studies[30]. All these studies were conducted in patients recruited from clinics and had small sample sizes relative to our pilot-study derived calculation of numbers needed for a powered trial of vitamin D intervention with IBS SSS as the outcome[31]. In view of this emerging literature and the potential benefit of vitamin D on IBS, coupled with the ease and relative safety of delivery we identified the need to assess the potential benefit of moderate dose vitamin D supplementation in the UK IBS population. Here we report on a double blind, placebo-controlled, adequately powered trial to investigate the effect of 12 week, moderate dose vitamin D supplement on symptoms of IBS. We hypothesised that vitamin D supplementation would reduce IBS symptom severity. This study was designed to test the hypothesis, and used a previous pilot study to inform the design [31].

## Materials and methods

### Study design

This was a randomised, double-blinded, placebo-controlled, two-arm parallel trial of 12-week duration. The study design and planned endpoints were registered at http://www.isrctn.com (ISRCTN13277340) seven weeks after recruitment had been initiated, but 11 months before trial closure or analysis. Ethical approval was granted by The University of Sheffield Medical School Research Ethics Committee (Ref: 11,865) and the trial was conducted in accordance with the Declaration of Helsinki. A sample size calculation (reported in our pilot study [31]) suggested, that 74 participants per arm were needed to achieve 80% power with 0.05 α–error (based on a reduction in total symptom severity score at exit of a mean of − 16 in the placebo arm, a mean of − 54 in the vitamin D intervention arm and a SD of 82). To achieve this target and allow for 10% withdrawals, a recruitment target of 160 participants was set.

### Participants and recruitment

Participants were recruited through online mailshots to volunteer lists through the University of Sheffield, via the IBS Network (The UK National charity for IBS) and through poster and postcard advertising in the local areas. Respondents were assessed according to trial criteria. The *Inclusion criteria* were: a previous clinical diagnosis of IBS by ROME criteria (as participation was open to individuals with longstanding IBS, potentially predating ROME IV or III, and as this was a community-based trial, of a potentially over-the-counter remedy, researchers required confirmation from participants of a previous clinical diagnosis, coupled to a total symptom severity score of 150 or over, rather than a clinical diagnosis using ROMEIV), age ≥ 18 years. *Exclusion criteria* were: regular use of nutritional supplements; pregnant or lactating; BMI > 30 kg/m^2^; BMI < 18 kg/m^2^; any history of other gastrointestinal disorders (e.g. inflammatory bowel diseases, diverticulitis, cancer); diabetes, recent or planned vacation. Due to circannual variation in vitamin D status[15] recruitment was undertaken seasonally in October–March 2017–18 and October–March 2018–19.

Respondents to advertisements were pre-screened against inclusion and exclusion criteria by telephone, provided with study information and subsequently invited to attend the Clinical Research Facility at the Royal Hallamshire Hospital, Sheffield for a study orientation and consent interview. At interview, potential participants’ inclusion/exclusion criteria were cross-checked, consent taken, BMI was measured, and the dosing and symptom reporting protocols were explained. Fortnightly symptom questionnaires (see below) were returned by post. Quality of life measures and blood spots for circulating 25(OH) vitamin D were taken at entry and exit interview.

Participants were provided with a sublingual flavoured liquid spray for delivery of 3,000 IU vitamin D3 *per diem*, and were instructed how to use the spray format. This trial is designed to support the option of self-administration / over the counter supplementation as an option for people with IBS. Dose was therefore selected to be (i) below the safe maximum daily dose [32]; (ii) effective at increasing circulating vitamin D in deplete subjects within the intervention period [33]. Placebo was an identically presented spray with vector and flavouring only. The vitamin D spray and identically packaged placebo were provided by BetterYou Ltd (Barnsley, UK). Randomisation was computer generated in blocks of eight using sealedenvelope.com by a third party (G. Weatherhead, BetterYou Ltd). Additional detail on the blinding process is in the online supplement (for additional detail see supplementary online material).

### Endpoints

Biometric data included age, sex, height (SECA 213 Height Measure), body weight (Tanita BC-543), circulating levels of vitamin D, severity of IBS and IBS-related Quality of Life. Participants’ circulating vitamin D was measured as 25(OH) vitamin D_2_ and 25(OH) vitamin D_3_ in a dry bloodspot using blood collected from a fingerprick blood sample at baseline and after 3 months on the intervention. The 25(OH)D assay was conducted by a clinical service provider (Black Country Pathology Services, Sandwell and West Birmingham NHS Trust) using a validated LC–MS-MS assay as previously described [33]. IBS symptoms were assessed every two weeks throughout the trial using a widely applied IBS symptom severity questionnaire [34]. The questionnaire scores both severity and duration of abdominal pain (Pain severity; days with pain), abdominal distension (Distension severity), satisfaction with bowel habits (bowel habit Satisfaction) and global well-being (Impact of symptoms on life). Scores for composite individual factors (each with an arbitrary score of 100) were combined to give the total Symptom Severity Score (SSS) which has a maximum value of 500. Participants were reminded to complete questionnaires and to continue to take vitamin D via fortnightly text messaging throughout the duration of the study. Quality of Life was assessed at baseline and exit using an IBS-specific QoL instrument [35]. Participants who completed the study received a £50 voucher to thank them for their time and effort.

### Data management and statistics

Consented participants were allocated consecutive trial numbers. The researcher (CEW) managed and inputted each participant’s biometric data, symptom severity scores and QoL data into a spreadsheet in SPSS v25.0 (IBM, Armonk, New York, USA). The standard duration of the intervention was 84 days.

Participants were advised to continue supplementation between day 84 and the exit meeting. “Days on trial” represents time from commencement to exit blood sampling, or to the day of the last recorded symptom questionnaire in the case of withdrawal.

**Fig. 1:**
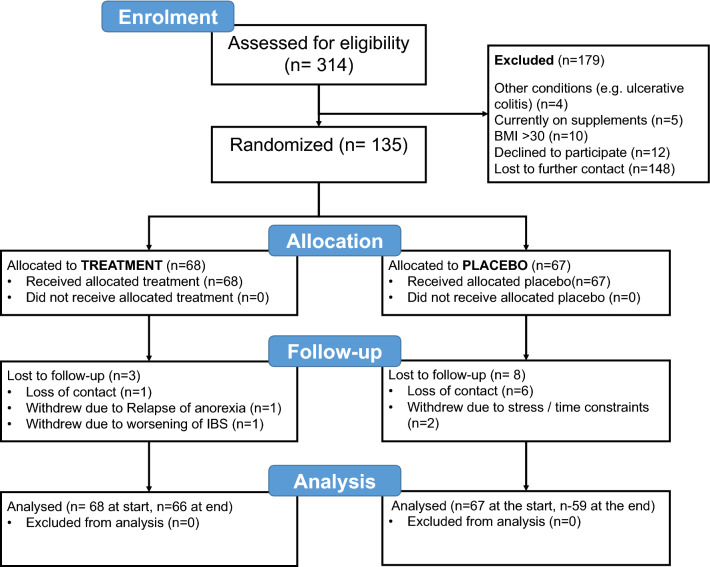
Consort diagram summarising participant recruitment and retention in this trial. Of 314 expressions of interest, 19 candidates did not meet the inclusion criteria, 10 declined further involvement and 148 did not follow-up on initial contact. 135 participants were entered into the trial; 92% were retained until scheduled exit, two were unable to meet the time commitment for involvement, one was for unrelated health reasons, one due to increased symptoms (not overtly framed as an adverse event by the participant) and seven lost contact

Data on serum 25(OH)D were returned to a third party (Mr G Weatherhead, BetterYou Ltd) who was blinded to all other participant data. Only on completion of the trial and data entry were spreadsheets merged. Analyses was undertaken by the research team whilst blinded to the identities of the trial arms. Analysis was performed on an intention to treat basis. Data missing for patients at the end of the trial period due to drop-out (see CONSORT diagram, Fig. 1) were not imputed. Statistical analyses were performed using SPSS V 25.0. Baseline demographic data were tested for normality and differences tested by t-test except where indicated; the primary endpoint (Symptom Severity Score) and contributing variables were analysed using repeated measures ANOVA. Non-normally distributed data are presented as medians with interquartile ranges and analysed by Mann–Whiney *U* test.

## Results

### Recruitment and patient demographics

Participants were recruited to this trial across two successive winters (2017–2018 and 2018–2019). In total, 135 participants were recruited from an initial 314 responses to trial publicity, with 179 either excluded or lost to contact prior to consent (see Fig. 1 for the CONSORT workflow). In total 80 participants were recruited in the 2017–2018 round and 55 in the 2018–2019 round. Sixty-eight participants were entered into the treatment arm and 67 received placebo; 92.5% of participants completed the trial, reasons for withdrawal are indicated where known. Only one participant (in the treatment arm) withdrew reporting worsened symptoms. Demographic data for the whole group and comparison of trial arms are shown in Table 1.

**Table 1:** Participant demographics at baseline

	All	Placebo	Treatment	*p*
Participants *n*	135	67	68	
Females *n* (%)	106 (78.5%)	51 (76.1%)	55 (80.9%)	0.5^a^
Age year	30.01 (± 10.46)	31.10 (± 10.85)	28.94 (± 10.03)	0.231^b^
BMI kg/m^2^	23.37 (± 2.88)	23.58 (± 3.00)	23.15 (± 2.76)	0.390^b^
IBS-SSS	277.41 (± 65.15)	273.22 (± 69.01)	281.54 (± 61.34)	0.460^b^
IBS-QoL %	42.72 (± 18.17)	43.35 (± 19.24)	42.54 (19.45)	0.809^b^
Blood 25(0H)D nmol/l (baseline)	49.23 (± 27.38)	49.71 (± 27.05)	48.75 (± 27.91)	0.839^b^
% with blood 25(OH)D < 50 mmol/l	60	61.2	58.8	0.779^a^
% with blood 25(OH)D < 25 mmol/l	20.7	14.9	26.5	0.098^a^
Dietary vitamin D intake µg/day (baseline)	3.09 (2.379)	3.21 (2.383)	2.96 (2.389)	0.565^b^

Data are summarised for the whole sample and by trial arm, where appropriate means (± SD) are listed, for days on trial medians (IQR) are shown. There were no between arm differences between any factor

^a^*χ*^2^ test

^b^*t* test

There were no differences between trial arms at baseline in proportion of females, mean IBS severity, mean IBS-related quality of life or serum 25(0H)D. In common with previous studies we found a high proportion of participants with IBS were below recommended vitamin D adequacy levels: 20.7% were deficient (< 25 nmol/l) and 60% were insufficient (< 50 nmol/l). Dietary intake of vitamin D was assessed at baseline, intake was 3.1 ± 2.38 µg/day in the study sample and there was no difference in intake between arms (Table 1).

### Effect of vitamin D supplementation on vitamin D status and IBS Symptoms

The intervention was effective at elevating total 25(OH)D levels, increasing circulating vitamin D in the intervention arm at 12 weeks relative to control (94.29 ± 33.70 *vs* 53.59 ± 23.21, *p* < 0.0001, *t* test) and relative to baseline (94.29 ± 33.70 vs. 48.75 ± 27.91, *p* < 0.001, t test). Exploratory analyses showed that the increase in circulating vitamin D in response to vitamin D intervention was greater in participants with insufficient vitamin D status (> 50 nmol/l) at baseline versus their replete counterparts (increasing by 56.1 ± 27.48 nmol vs 30.0 ± 34.1 nmol, p = 0.001) and also greater for those participants who were deficient (> 25 nmol/l) at baseline (increasing by 60.1 ± 31.02 nmol vs 40.1 ± 32.26 nmol, *p* = 0.034) (Table 2).

**Table 2:** Outcome measures

Outcome	Placebo	Treatment	*p*
Adverse events	2	2	
Days on Trial (IQR)	83 (15)	85 (11)	0.240^a^
IBS-SSS (Baseline)	273.22 (± 69.01)	281.54 (± 61.34)	0.460
IBS-SSS (Exit)	195.37 (± 97.27)	220.32 (± 93.72	0.147
IBS-QoL % (Baseline)	43.64 (± 18.33)	41.81 (± 18.09)	0.560
IBS-QoL % (Exit)	33.12 (± 17.95)	34.24 (± 17.56)	0.726
Blood 25(0H)D nmol/l (baseline)	49.71 (± 27.05)	48.75 (± 27.91)	0.839^b^
Blood 25(0H)D nmol/l (exit)	53.59 (± 23.21)	94.29 (± 33.70)	< 0.0001^b^

Data are comparisons by trial arm; where appropriate the means (± SD) are listed, for Days on Trial medians (IQR) are shown. There were no between arm differences for the primary outcome measure (IBS-SSS) or QoL. There was a significant difference between trial arms in circulating vitamin D at trial exit (*p* < 0.0001

^a^Mann–Whitney *U* test

^b^*t* test

**Fig. 2:**
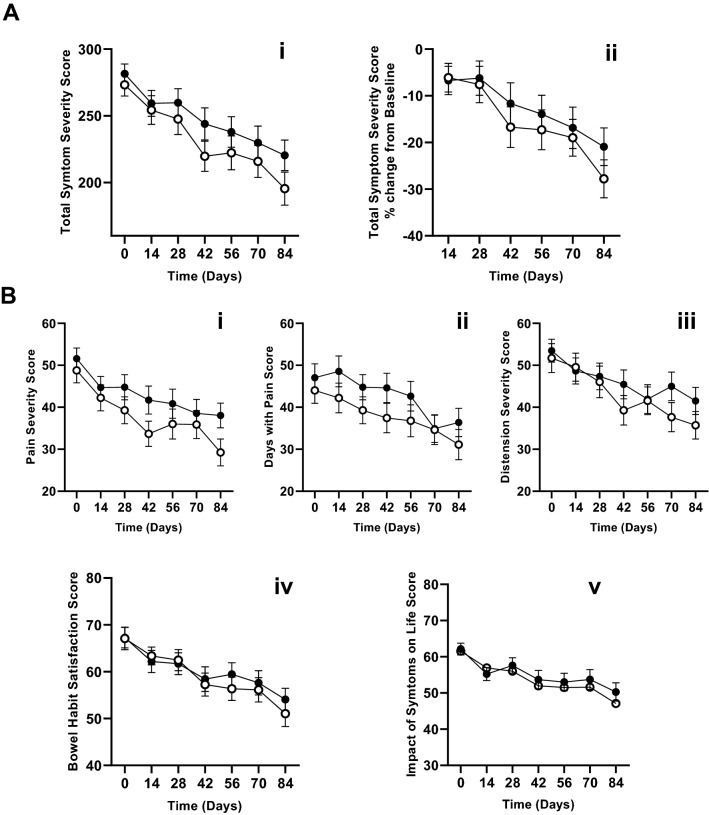
Effect of vitamin D supplementation on IBS symptoms. Participants were assessed every 2 weeks on their symptoms. In all plots, placebo arm is the open circle and active arm is the solid circle; plots show mean ± SEM at each timepoint. **A** Shows change in total symptoms across the course of the trial, Panel Ai shows actual symptom severity, Panel Aii shows change from baseline. **B** Shows each symptom score plotted in the same way. I–iv are, respectively, pain severity, days with pain, distention severity, satisfaction with bowel habit and affected life

The primary outcome measure was IBS-SSS. To assess the effect of vitamin D on IBS symptoms, the symptom severity was assessed every 2 weeks across the course of participation. Analysis of total symptom severity over time by trial arm is shown in Fig. 2Ai. Both groups reported significant improvement in their IBS symptoms, but there was no difference between vitamin D and placebo treatment arms (*p* =  0.824, ANOVA). The data were also considered as change from baseline (Fig. 2Aii) and again no difference was identified between the trial arms (*p* = 0.872, ANOVA). The IBS-SSS was compared at the 12 week point (see Table 1). At this timepoint, there was no difference between trial arms in total symptom severity (Vit D = 220.3(± 93.73), vs Placebo = 194.2 (± 97.67) *p* = 0.147). When individual symptom scores were assessed (Severity of pain, days with pain, distention, satisfaction with bowel habit, and impact of symptoms on life) there were no differences between trial arms across the course of the study for any individual symptom (data for all timepoints are provided in the Supplementary material). No differences in response to the intervention were identified according to IBS subtype (data not shown).

Response to intervention may be dichotomised; a reduction in symptom severity of more than 50 points is invoked as clinically effective/ beneficial [34]. When proportions of participants exhibiting > 50point were compared for treatment vs. placebo (Table 3), there was no difference in response rate between arms.

**Table 3:** Comparison of response rate between trial arms

	Frequency (%)	*p*
All Participants		
Placebo	38/60 (63.3%)	
Treatment	37/65 (56.9%)	0.465
Vitamin D insufficient/deficient participants (25(OH)D < 50 nmol/L)		
Placebo	22/36 (61.1%)	
Treatment	20/37 (54.1%)	0.542
Vitamin D deficient participants ((25 (OHD) < 25 nmol/L)		
Placebo	5/8 (62.5%)	
Treatment	8/15 (53.3%)	0.673

Response is defined as > 50 point reduction in TSS score at trial exit. There were no differences in the proportions of participants responding to the intervention by trial arm in the whole study, or in either lower vitamin D status category (inadequate and deficient, deficient) (χ^2^ test)

**Fig. 3:**
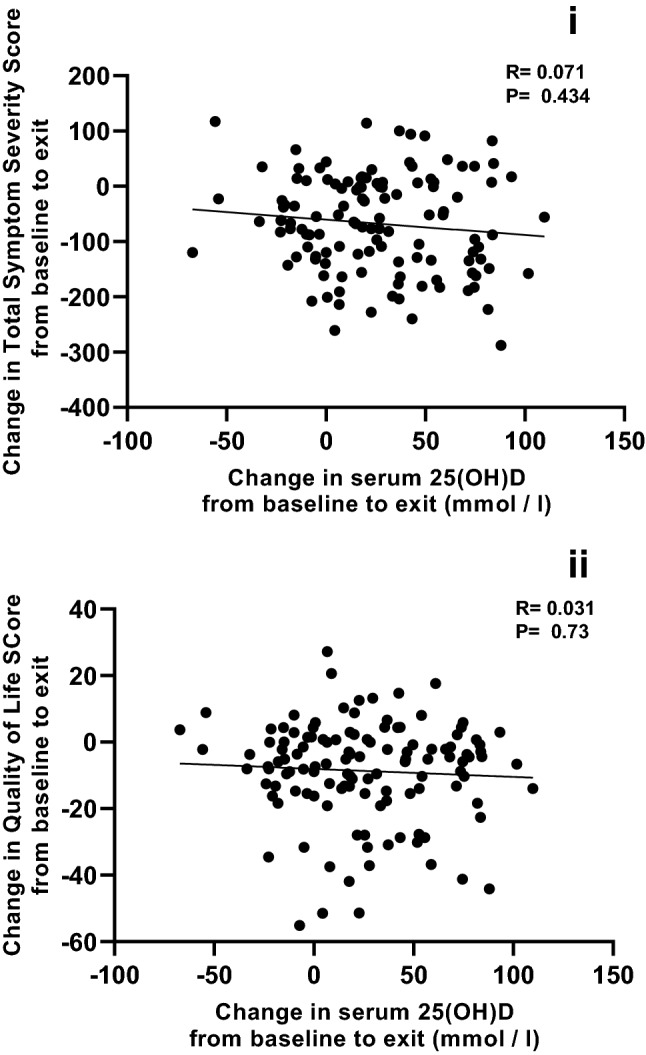
Effect of change in vitamin D status on IBS symptoms and quality of life. The effect of change in circulating levels of vitamin D was assessed for both outcome measures (TSS and QoL). **a** shows correlation between change in circulating vitamin D from start to end of the trial against change in IBS symptoms. **b** shows correlation between change in circulating vitamin D from start to end of the trial against change in Quality of Life. There was no relationship between either endpoint and the vitamin D status change (Spearman’s rank correlation coefficients shown)

Finally, we hypothesised that the extent of improvement in circulating vitamin D level might lead to improvement in symptoms and tested this by correlating change in vitamin D with change in symptoms. There was no apparent relationship between change in serum 25(OH)D and change in total symptom severity (Fig. 3i; *r* = − 0.071, *p* = 0.434, Spearman’s rank correlation coefficient).

### Effect of vitamin D status on quality of life in IBS

Several studies have used an IBS-specific QoL instrument [36] and reported a benefit of vitamin D intervention. The instrument was applied at baseline and at exit from the intervention. Whilst there was an improvement in QoL in each arm of the trial (*p* < 0.001 for each arm, Mann–Whitney), there was no difference between the change in QoL score from baseline to exit between trial arms (*p* = 0.525, Mann–Whitney). We investigated whether improvement in circulating vitamin D level might improve QoL; no relationship was found between change in serum 25(OH)D and change in QoL (Fig. 3ii; *r* = − 0.031, *p* = 0.73, Spearman’s rank correlation coefficient).

### Exploratory and signal-seeking analyses

Trials in IBS often either select or subdivide participants according to IBS subtype (constipation, diarrhoea or alternating symptoms). A signal seeking analysis was undertaken to assess whether there were differences in response to vitamin D by IBS Subtype. There was no difference in symptom severity (SSS: *p* = 0.719, 0.962, 0.697 constipation, diarrhoea and alternating symptoms, respectively, Repeated measures ANOVA) or change in Quality of life (QoL *p* = 0.316. 0.946, 0.090 constipation, diarrhoea and alternating symptoms, respectively, Mann–Whitney *U* test) in response to vitamin D within any of the IBS subtype groups.

The response according to IBS severity was investigated. Participants were categorised by IBS severity[34] (75–174—Mild; 175–299—Moderate; > 300—Severe) and response to the intervention was analysed. There were no differences in symptom severity (*p* = 0.25, 0.518, 0.554 mild, moderate and severe, respectively, repeated measures ANOVA) or Quality of life (*p* = 0.262. 0.275, 0.900 mild, moderate and severe, respectively, Mann–Whitney *U*) in response to intervention when analysed according to IBS symptom severity at baseline.

## Discussion

This study sought to investigate the potential of vitamin D supplementation as a management strategy for IBS, the design was community-based, seeking to be applicable to the general IBS population in addition to clinical settings. This study found no benefit of vitamin D supplementation on either symptoms of IBS or on QoL measures using standardised assessments. In addition, we found no relationship between change in vitamin D and change in symptomology.

The study has several hallmark features: it was based on a formal pilot study using the same intervention, endpoints and population for the full trial; it is the largest trial of vitamin D in people with IBS; it used a moderate and safe dose of vitamin D; the duration of intervention was determined to minimise placebo effect [30]. Due to circannual variation in vitamin D status, we undertook recruitment during the winter to potentiate the maximum increase in circulating vitamin D at the annual low, concomitantly minimising risk of reaching toxic levels of the vitamin. Limitations of our trial include the potential heterogeneity of the sample (although this was deliberately a real-world study). We may have achieved more sample homogeneity and reinforced IBS diagnosis through reassessing participants with the ROMEIV criteria at screening. This sample would be more homogenous, although not necessarily more responsive. A general risk in nutrient supplement trials is that patients may self-supplement, obscuring effects; this was minimised by analysing outcomes against change in circulating vitamin D as well as by trial arm. We did not meet our target sample size, based on the power calculation. The implementation of GDPR regulations in 2018 led to a substantial impact on our recruitment rate in the second winter (80 vs target of 80 in first season; 55 versus target of 80 in second season). The absence of any signal of an effect suggests that failure to recruit did not affect interpretation of the outcome. Finally, despite our design, the placebo effect remained large.

Our findings are in contrast to a cluster of recent trials reporting a benefit of vitamin D supplementation on symptoms of IBS[25–28]. Abbasnezhad et al*.*[25] based in Iran recruited 45 outpatients / arm to a 50,000 IU fortnightly dose for 21 weeks and reported a significant reduction in symptoms (*p* < 0.001) of over 70 TSS points on average. Jalili et al.[26] had only 25 patients/arm recruited from an endoscopy clinic in Iran to 50,000 IU “biweekly”^1^ dose for 6 weeks, again reporting a significant (*p* < 0.05) response. El Amrousy et al. [28] had a larger sample size (56/arm) recruited from paediatric outpatients in Egypt, undertook a power calculation based on a vitamin D intervention in IBS,^2^ and used a longer intervention (21 weeks), again finding a significant (*p* < 0.001) benefit of supplementation. Jalili et al. [27] (2019) again recruiting in Iranian endoscopy clinics and using a dose (50,000 IU p.w.) considerably in excess of what would be regarded as safe, for 6 weeks with 58 patients per arm, again found a significant (*p* < 0.05) benefit of vitamin D. Most recently Sikaroudi et al. [29] recruited 88 patients from a gastroenterology clinic, dosing with 50,000 IU p.w. for 9 weeks, and reported a significant improvement in IBS-SSS. A further publication from the same group appears to be a restatement of these outcomes[37]. We note that these trials have several consistent features that limit their generalizability—all are based on clinically recruited groups in the Middle East; three used an intermittent bolus dose (50,000 IU), with one study using an extremely high effective dose of 7,142 IU *p.d*. Nonetheless, all four studies reported high compliance, low rates of drop out and high levels of significance notwithstanding sample sizes (25–58/arm) which our power calculation suggests were small. Despite the success of these trials their features suggest caution is needed about generalisability of their findings to the wider IBS population; in particular a bolus dose of 50,000 IU would not be a recommendable approach for general symptom management in IBS.

A recurrent feature of IBS trials is the heterogeneity of response, which may in part reflect the ill-defined nature of the syndrome. A meta-analysis of coefficients of variation (CV) in the IBS symptom tool used in this study reveals an average CV of 25% (SD = 8%) (Williams & Corfe, manuscript in preparation). It may be the case that there are subsets of the IBS population who do benefit from vitamin D supplementation[23]. Predicting responders, in terms of IBS symptoms, merits further research as vitamin D supplementation is a viable long-term management option. Our work shows that neither vitamin D status nor repletion is a predictor of a therapeutic response to vitamin D supplementation (in contrast, for example, to IBD[38]). Exploratory analyses of larger datasets would be needed to identify such potential predictors.

Critically, this study is in line with others in identifying vitamin D deficiency as widespread in IBS. There is recent, increasing recognition that IBS associates with increased risk of fracture[39] and of osteoporosis[40]. A causal inference is not yet possible, but poor vitamin D status in IBS may contribute to the observed association of these conditions. This suggests that, notwithstanding any benefit of vitamin D on IBS symptomology, IBS patients should be screened for vitamin D status and supplemented appropriately for general health reasons.

## Supplementary Information

Below is the link to the electronic supplementary material.

Supplementary file1 (DOCX 21 kb)

## Data Availability

Anonymised spreadsheets will be made available on reasonable request to the corresponding author.

## Footnotes

1 It is unclear whether “biweekly” meant twice weekly or fortnightly in the context of this paper.

2 Although the calculation appears to be based on work published after the group started recruiting.
