# Supplementary material for: Vitamin D supplementation in people with IBS has no effect on symptom severity and quality of life: results of a randomised controlled trial
Source: Eur J Nutr. 2021 Jul 30;61(1):299–308. doi: 10.1007/s00394-021-02633-w (PMC8783891; doi:10.1007/s00394-021-02633-w)
Supplement: Supplementary file 1 — Supplementary file1 (DOCX 21 kb) [file 394_2021_2633_MOESM1_ESM.docx]

**Supplementary Information for:**

**Vitamin D supplementation in people with IBS has no effect on symptom severity and quality of life: results of a randomized controlled trial**

Claire E. Williams^1^, Elizabeth A. Williams^2^ & Bernard M. Corfe^1,3^

1. Molecular Gastroenterology Research Group, Department of Oncology & Metabolism, The University of Sheffield, The Medical School, Beech Hill Road, Sheffield, S10 2RX, UK
2. Healthy Lifespan Institute, Department of Oncology & Metabolism, The University of Sheffield, The Medical School, Beech Hill Road, Sheffield, S10 2RX, UK
3. Population Health Sciences Institute, Human Nutrition Research Centre, Faculty of Medical Sciences, Newcastle University, Newcastle, NE2 4HH

*Author for correspondence:* Prof Bernard Corfe, Population Health Sciences Institute, Human Nutrition Research Centre, Faculty of Medical Sciences, Newcastle University, Newcastle, NE2 4HH; email: [bernard.corfe@newcastle.ac.uk](mailto:b.m.corfe@gmail.com)

**Supplementary Information 1: Randomisation strategy**

The randomisation schedule was generated independently by G. Weatherhead (BetterYou Ltd) using sealedenvelope.com and a block size of eight. Identically presented boxes containing coded bottles and corresponding coded vitamin D bloodspot tests were provided to the research team by the supplier. The research team (CEW, EAW, BMC) was blinded to the content of each bottle.

In addition the vitamin D assay results were returned and collated by a third party (G. Weatherhead, BetterYou) who was blinded to the IBS-SSS scores, whilst the researcher (CEW) entered the IBS-SSS data into a parallel sheet, blinded to the vitamin D baseline status or change. As the trial took place over a long period, across two successive winters, this protocol ensured that no interim unblinding or analyses were undertaken until all the data entry was completed.

**Supplementary Information #2 – Data Table for IBS-SSS outcomes**

|  |  | **Time (days)** | | | | | | | |
| --- | --- | --- | --- | --- | --- | --- | --- | --- | --- |
|  |  | 0 | 14 | 28 | 42 | 56 | 70 | 84 |  |
| **Pain severity†**  **mean (SD)** | Vit D | 51.6 (21.20) | 44.7 (21.63) | 44.8 (24.64) | 41.7 (27.35) | 40.9 (28.10) | 38.6 (26.43) | 38.1 (23.91) |  |
|  | Placebo | 48.8 (24.59) | 42.3 (25.23) | 39.29 (26.16) | 33.7 (24.05) | 36.0 (28.44) | 35.9 (25.74) | 29.2 (24.82) |  |
|  |  |  |  |  |  |  |  |  |  |
| **Days with pain†**  **mean (SD)** | Vit D | 47.1 (27.58) | 48.6 (30.07) | 48.3 (28.57) | 44.6 (28.53) | 42.7 (28.52) | 34.9 (26.13) | 36.4 (27.35) |  |
|  | Placebo | 44.0 (25.47) | 42.2 (28.75) | 43.7 (29.73) | 37.4 (28.01) | 36.8 (30.19) | 34.6 (27.15) | 31.1 (27.93) |  |
|  |  |  |  |  |  |  |  |  |  |
| **Distension severity†**  **mean (SD)** | Vit D | 53.4 (22.69) | 48.6 (25.38) | 47.3 (26.07) | 45.5 (28.25) | 42.0 (27.78) | 45.0 (26.72) | 41.5 (26.31) |  |
|  | Placebo | 51.7 (28.46) | 49.6 (26.99) | 46.1 (30.66) | 39.27 (28.1) | 41.5 (26.27) | 37.7 (26.77) | 35.7 (25.28) |  |
|  |  |  |  |  |  |  |  |  |  |
| **Bowel Satisfaction†**  **mean (SD)** | Vit D | 67.3 (18.37) | 62.2 (19.41) | 61.7 (19.23) | 58.4 (21.54) | 59.4 (19.99) | 57.6 (20.36) | 54.1 (19.43) |  |
|  | Placebo | 67.1 (19.60) | 63.4 (15.49) | 62.5 (18.51) | 57.3 (19.86) | 56.4 (19.98) | 56.1 (19.94) | 51.0 (21.45) |  |
|  |  |  |  |  |  |  |  |  |  |
| **Impact on Life†**  **mean (SD)** | Vit D | 62.1 (13.64) | 55.2 (14.94) | 57.6 (17.57) | 53.7 (20.97) | 53.0 (19.71) | 53.7 (21.60) | 50.3 (20.8) |  |
|  | Placebo | 61.6 (14.93) | 57.0 9 (17.15) | 56.0 (20.39) | 52.0 (19.71) | 51.5 (20.83) | 51.6 (21.67) | 47.1 (22.96) |  |
| **Total-SSS**  **mean (SD)** | Vit D | 281.54 (61.33) | 259.3 (79.26) | 259.8 (86.15) | 243.9 (99.17) | 237.9 (92.06) | 229.79 (98.40) | 220.3 (93.73) |  |
|  | Placebo | 273.2 (69.01) | 254.4 (87.48) | 247.5 (93.80) | 219.6 (90.78) | 222.2 (100.86) | 215.9 (92.95) | 194.2 (97.67) |  |

**Table S1 Data detail for response to treatment**

The table shows mean and standard deviations for each composite IBS symptom and for the total Symptom Severity Score at each timepoint in the study for each arm of the study, supporting the data presented graphically in Fig 2. Individual scores have a maximum score of 100; Total SSS _: Total symptom severity has a maximum possible score of 500.
